# BMI and specimen weight: impact on personalized risk profiling for optimized informed consent in breast reduction surgery?

**DOI:** 10.1038/s41598-019-49169-y

**Published:** 2019-09-03

**Authors:** Raimund Winter, Frederike M. J. Reischies, Alexandru Tuca, Paul Wurzer, Christian Schubert, Christina H. Wolfsberger, Theresa Rienmueller, Herwig Friedl, Michaela Sljivich, David B. Lumenta, Lars-P. Kamolz

**Affiliations:** 1 Division of Plastic, Aesthetic and Reconstructive Surgery, Department of Surgery, Medical University of Graz, Auenbruggerplatz 5, A-8036 Graz, Austria; 2 Institute of Health Care Engineering with European Notified Body of Medical Devices, Graz University of Technology, Stremayrgasse 16, A-8010 Graz, Austria; 3 Institute of Statistics, Graz University of Technology, Kopernikusgasse 24, A-8010 Graz, Austria; 4 University of Texas Medical Branch, School of Medicine, 301 University Blvd, Galveston, TX 77555 USA; 5 COREMED – Cooperative Centre for Regenerative Medicine, Joanneum Research GmbH, Neue Stiftingtalstr. 2, A-8010 Graz, Austria

**Keywords:** Outcomes research, Risk factors

## Abstract

We aimed to evaluate the interaction between individual risk factors and institutional complication rates after reduction mammaplasties to develop a chart for a personalized written patient informed consent. We retrospectively reviewed charts of 804 patients who underwent bilateral breast reduction between 2005 and 2015. The Clavien-Dindo classification was used to classify postoperative complications. Relevant predictors were found by applying a stepwise variable selection procedure. Multilevel predictors were assessed through chi-square tests on the respective deviance reductions. 486 patients were included. The most common complications were wound healing problems (n = 270/56%), foreign body reactions (n = 58/12%), wound infections (n = 45/9, 3%) and fat tissue necrosis (n = 41/8%). The risk factors for the personalized patient chart for the most common complications influencing the preoperative informed consent were: smoking, operative technique, resection weight for wound healing problems; body mass index and allergies for wound infections; and patients’ age, resection weight for fat tissue necrosis. The resultant chart of institutionally encountered most common complications based on individual risk factors is a graphical template for obtaining patient informed consent in the future. Whether this approach influences patient information retainment, incidence of filed lawsuits or behavioral change needs to be prospectively tested in future studies.

## Introduction

Women with large breasts, macromastia or even gigantomastia suffer from symptoms such as neck and back pain possibly leading to chronic headaches and range of motion impairment^1^. In some cases, large breasts can also cause mastopathy and even ulceration of the skin in the inframammary fold^2,3^. Besides the physiological burden, large breasts can result in^4^ a severe psychological distress; and affected women are often confronted with social struggles ranging from being overly sexualized to troubles in finding a partner for a relationship^5^. Reduction mammaplasty can decrease these patients’ burden and significantly improve their quality of life^6^.

Postoperative complication rates after reduction mammaplasty vary between 4% and 63%; and common complications include wound healing problems, wound infections, fat tissue necrosis or foreign body reactions^3,7–13^. Beside this high complication rates and the fact that some complications are more likely to develop^13^, patients can only be informed based on the operation and associated complications of published percentages, but not on their individual risk profile. In general, patients usually remember just 3 out of 12 common complications following oral and written informed consent^14^. Studies have suggested that certain risk factors are associated with specific postoperative complications: especially an elevated resection weight and an as obese classified body mass index (BMI) have significant impact on wound healing problems^15^.

The precise interactions of multiple risk factors in relation to the BMI or the resection weight have so far not been investigated. This knowledge is key for an optimized informed consent and we aimed to evaluate the interaction between several risk factors and intraoperative interventions on postoperative complications to create an image-based chart to aid in obtaining informed consent prior mammaplasty.

## Methods

We retrospectively reviewed charts of 804 patients aged between 18 and 81 years, which underwent a bilateral reduction mammaplasty at our institution between 2005 and 2015 (inclusion criteria). The study protocol was approved by the institutional review board with a waiver for acquiring further informed consent due to the retrospective nature of the analysis (EK-Nr.1175/2015). Exclusion criteria were: breast cancer in the past medical history, a prior breast operation, treatment with any systemic immunodeficiency of immunosuppressive medications. All reduction mammaplasties were performed by plastic surgeons and under general anesthesia. Intraoperatively, a drain was applied in each breast and they were removed when the 24 hr drainage was below 50cc. Patients visited the outpatient clinics for routine inspection at 2 weeks, as well as 3, 6 and 12 months postoperatively.

The data was retrieved from the clinical and operative notes, patient charts, nursing reports and anesthetic protocols. The gathered data was divided into patient-specific, treatment-specific and complication-specific data. Patient-specific data included weight, age, smoking, diabetes mellitus, allergies, BMI and hypertension. The treatment-specific information was preoperative single dose antibiotics, duration of surgery (defined as the time between tracing the lines of incision to the completion of the last suture), drain output, time to drain removal, reduction weight, surgical technique, fluid balancing and in-hospital revision surgery before discharge. Complication-specific data collected were wound healing problems, wound infections, fat tissue necrosis, seroma, hematoma, late seroma and foreign body reactions. The Clavien-Dindo classification^16^ was used to classify postoperative complications.

The statistical analysis was performed with R 3.4.0^17^, a free software environment for statistical computing and graphics. Relevant predictors (influential variables or multilevel factors) in the logistic models were found by applying a stepwise variable selection procedure. Predictors were finally tested for significance by two-sided t-tests. Multilevel predictors were assessed through chi-square tests on the respective deviance reductions. P-values below 0.05 were considered statistically significant.

### Statement of informed consent

Consent was obtained from all individual participants included in the study.

### Statement of human and animal rights

The protocol of this analysis was approved by the Institutional Review Board of the Medical University of Graz and was in accordance with the Helsinki Declaration of 1975 (revised 2000). References

## Results

Four hundred and eighty-six patients met the inclusion criteria (Table 1). The analysis of the operative technique showed five different groups in decreasing order: the wise pattern technique (WPT) with a superior pedicle (77%), the vertical technique with a superior pedicle (11%), WPT with inferior pedicle (7%), other techniques (3%) and WPT with free nipple graft (2%).

**Table 1:** General patient information.

*Patient-specific data*	Mean (+/− SD)	Number/Percentage
Weight	71.7 kg (+/−10.4)	
Age	39 years (+/−12)	
BMI	26.4 kg/m^2^ (+/−3.56)	
Smoking		170/34.9%
Diabetes mellitus		7/1.4%
Allgeries		208/42.8%
Hypertension		66/13.6%
***Treatment-specific data***		
Preoperative single dose antibiotics		399/82.1%
Duration of sugery	160 min (+/−47)	
Drainage output	143 ml (+/−115)	
Length of drainage stay	4 days (+/−1)	
Weight of reduction specimen	1168 g (+/−579)	
Interoperative fluid input/output	3198 ml (+/−937)	
Interoperative fluid output	779 ml (+/−491)	
Revision sugery (medical and aesthitical reasons)		58/11.9%
***Complication-specific data***		**Number/Percentage**
Wound healing problem		270/55.6%
Infections		45/9.3%
Fat tissue necrosis		41/8.4%
Seroma		6/1.2%
Late seroma (>14d)		6/1.2%
Haematoma		14/2.9%
Foreign body reaction		58/11.9%
***COMPLICATIONS (Clavien-Dindo classification)***		**Number/Percentage**
Grade I		234/48%
Grade II		46/9%
Grade IIIa		2/<1%
Grade IIIb		22/5%
Grade Iva		0/0%
Grade IVb		0/0%
Grade V		0/0%

Logistic models with patient-, treatment- and complication-specific predictors demonstrated for four complications statistically significant findings: wound healing problems, wound infections, fat tissue necrosis and foreign body reactions (Table 2).

**Table 2:** Predictors.

wound healing problems	
operative technique	*p* = *0*.*0009*
administration of preoperative single dose antibiotics	*p* = *0*.*001*
resection weight	*p* < *0*.*0001*
revision surgery	*p* = *0*.*012*
smoking	*p* = *0*.*037*
**infections**	
BMI	*p* = *0*.*0002*
administration of preoperative single dose antibiotics	*p* = *0*.*03*
allergies	*p* = *0*.*01*
**foreign body reaction**	
BMI	*p* = *0*.*04*
revision surgery	*p* = *0*.*039*
**fat tissue necrosis**	
surgical revision	*p* < *0*.*0001*
resection weight	*p* = *0*.*008*
administration of preoperative single dose antibiotics	*p* = *0*.*042*
age	*p* = *0*.*046*

### Wound healing problems (WP)

A wound healing problem was defined as an area of the wound, which did not heal *per primam intentionem* and left a gap in the continuity of the scar no matter how small. Overall, 56% of all patients had a gap in the continuity of the scar. Five significant predictors could be identified. These were the operative technique (p = 0.0009), the administration of preoperative single dose antibiotics (SDA) (p = 0.001), resection weight (p < 0.0001), revision surgery (p = 0.012), and smoking (p = 0.037). WPs were significantly related to the operative technique. The WPT with a cranial pedicle was used in most of the cases, therefore it defined the reference technique and the other ones were compared to it. For the vertical technique, the odds to develop a WP were 3.9 times higher as compared to a cranial pedicle. The model also showed that administration of preoperative SDA decreased the chance to develop a WP by 57%. If the weight of the resected breast tissue increased by 100 gram the chance for developing a WP increased by 18.4%. Revisions doubled the chance of WP, and smoking increased this chance by (another) 53%.

### Infections (INF)

An INF was defined as any sign of “redness, swelling, pain, or heat at the site of a wound” as well as raised inflammatory markers in the blood, which triggered the use of antibiotics. Nine percent of all patients got an INF after surgery. BMI (p = 0.0002), SDA (p = 0.03) and allergies (p = 0.01) were significant predictors for an INF. When the BMI was increased by one point, the chance to get an infection after RM was increased by 16.4%. Increasing the BMI by 5 or 10 units increased the probability by 114% and 358%, respectively. The SDA decreased the chance for an INF by 55% as compared to administering no SDA. Furthermore, we found that patients with allergies had a 2.3 times higher chance to develop a postoperative INF after RM than patients without any allergies.

### Foreign body reaction (FBR)

A FBR was any wound breakdown with exposed suture material no matter how small. Twelve percent of all patients had a FBR. BMI (one-sided p = 0.04) and revision surgery (p = 0.039) had a significant impact on its occurrence. By increasing the BMI by one, 5, and 10 units, the chance to develop an FBR increased by 7.1%, 40.7% and 98.1%, respectively. Moreover, the chance to get an FBR was 2.2 times higher after a revision surgery.

### Fat tissue necrosis (FTN)

A FTN was defined as the clinical presentation of hardened tissue in the breast after reduction mammoplasty, which was diagnosed by ultrasound or confirmed intraoperatively during revision. Overall 8.4% of all patients presented with a FTN. The four significant predictors were surgical revision (p < 0.0001), resection weight (p = 0.008), administration of preoperative single dose antibiotics (p = 0.042), and age (p = 0.046). The model reflects that after a revision surgery the chance to develop a FTN was 4.9 times higher than without one. The higher the resection weight by 100 grams increased the chance for a FTN by 15.1%. Furthermore, each single additional, five and 10 more years-of-age increased the chance for developing an FTN by 2.9%, 15.9% and 34.2%, respectively. The administration of preoperative single dose antibiotics reduced the chance for a FTN by 55.1%.

For the patient-specific data seroma, late seroma and hematoma the number of cases was not sufficiently large enough to allow a reasonable estimation of the respective logistic regression models.

All complications were classified according to the Clavien-Dindo classification and assigned to the following degrees: 48% Grade I complications, 9% Grade II, <1% Grade IIIA and 5% Grade IIIB.

### Patient chart

**Figure 1:**
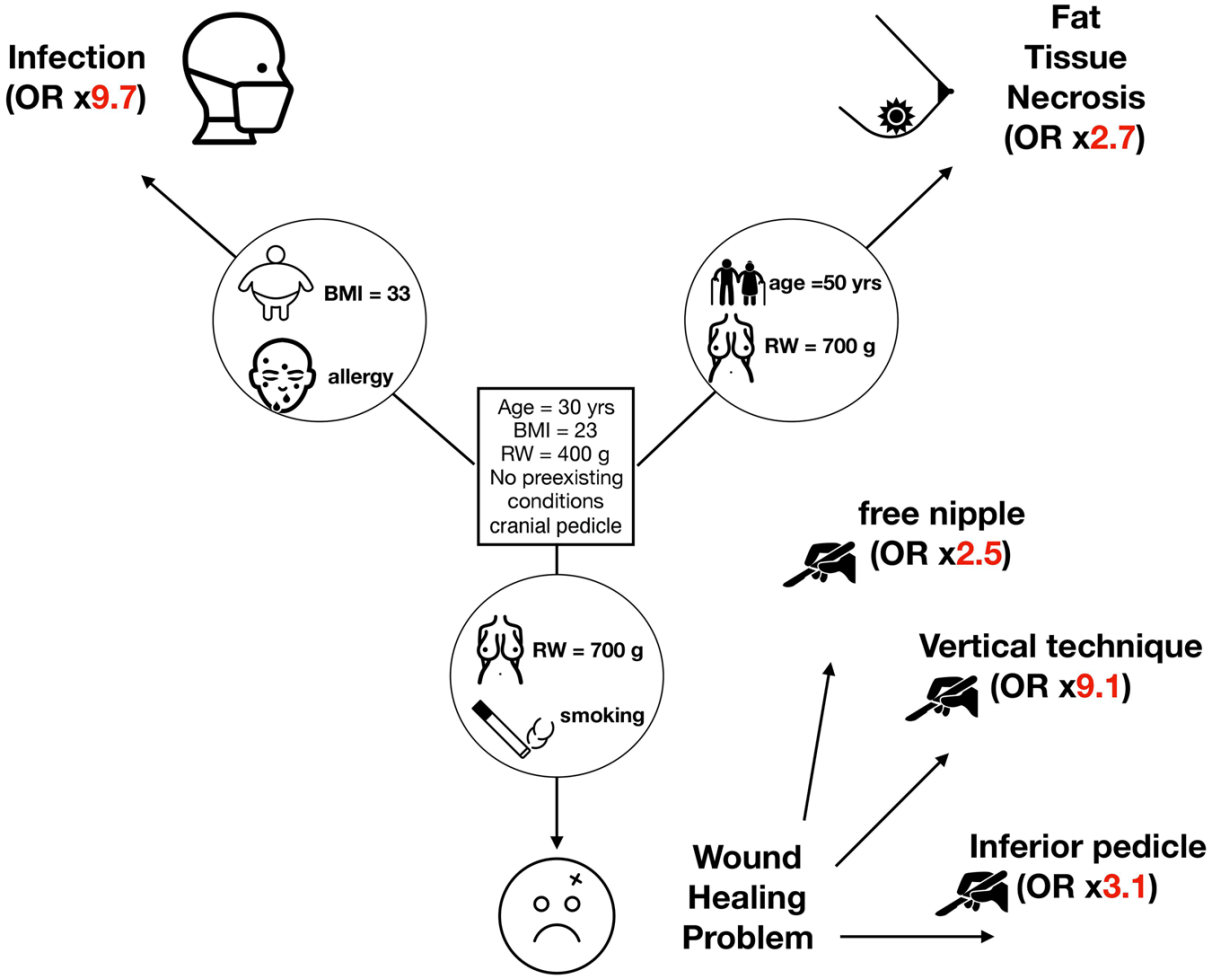
Shows the interaction between patient characteristics (30 years, BMI 23, resection weight 400 g, no preexisting conditions, cranial pedicle) and risk factors, which lead to increased chance of post-operative complications (infection, fat tissue necrosis, wound healing problems). OR = odds ratio.

**Figure 2:**
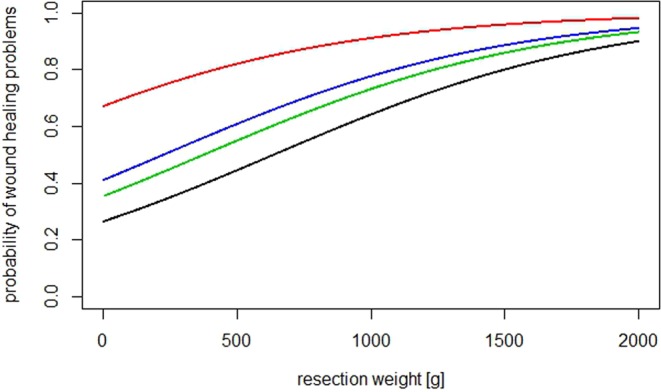
Shows the estimated probabilities for WP in a smoker with 700 g resection weight (75% Quantil). Black line: reference*; blue line: smoker, resection weight of 700 g and inferior pedicle; green line: smoker, 700 g resection weight and free nipple; red line: smoker, 700 g resection weight and vertical technique.

**Figure 3:**
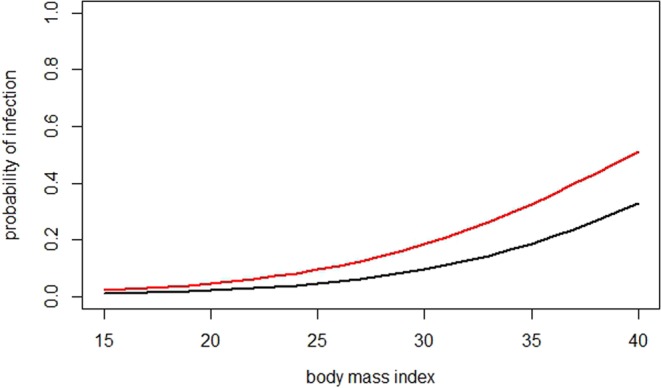
Shows the estimated probabilities for INF under the logistic model depending on all significant predictors. Black line: reference*; red line: patient with allergies and BMI 33.

**Figure 4:**
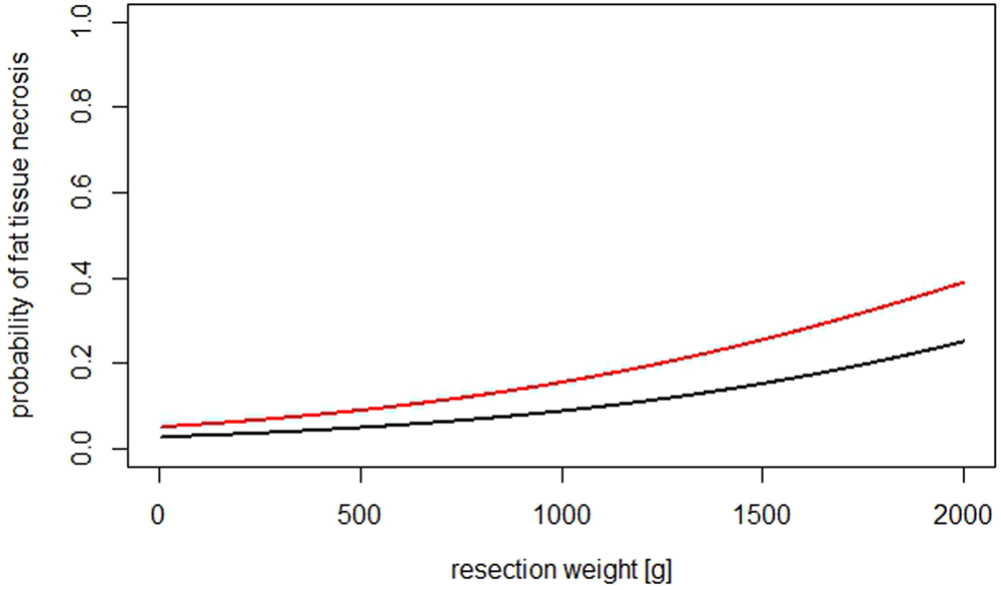
Shows the behavior of the model for FTN and its dependence on the predictors. Black line: reference*; red line: 50 years of age, and resection weight of 700 g. * 30 year-old non-smoker with BMI of 23, no allergies, and a resection weight of 400 g.

To better illustrate the above findings, we created a two-sided patient chart to provide an illustrative guide for preoperative informed consent in our institution. Figure 1 shows the patient chart’s frontside with an illustration to simplify the risk factors, the backside contains the 3 logistic regression models (Figs 2–4), by applying a ruler on the two respective axes an OR estimate for most common complications based on an individual precondition can be obtained. For this patient-chart model, we omitted treatment specific predictors like the administration of preoperative single dose antibiotics or revision surgery from the analysis, which were factors falling outside a patient’s sphere of influence during the preoperative informed consent.

In our patient-chart model, smoking (p = 0.044), operative technique (p = 0.001), and resection weight were statistically significant predictors for WP. Furthermore, BMI (p = 0.0002) and allergies (p = 0.020) were significant predictors for an INF; patients’ age (p = 0.017) and resection weight (p = 0. 018) were significant predictors for FTN. There were no statistically significant predictors for FBR in the patient-chart model.

## Discussion

To the best of our knowledge this is the first work based on prospectively collected data analyzed by a multivariate logistic regression model, which provides a simplified patient chart for stratifying the odds for developing the most common complications after a specific operation depending on an individual’s pre-existing conditions.

These data provide a unique opportunity for explaining complications and giving informed consent to patients, since probabilities are based on individual risk factors, facilitating the practice approach in an individualized manner. There is varying body of evidence for the risk factors detected in our analysis for or against its possible associations in comparison to the current literature. In fact, there is less focus on the combination of common or severe complications, and even less so on associations with pre-existing conditions and their attributable factoring for developing complications in pooled datasets or single/few center analyses. Therefore, attempts to compare all data based on published analyses in the medical literature are quite challenging and can become biased, if only interpreted by use of a single complication.

Beginning with increasing resection weights and complications after RM some (single center) literature sources found an association^3,12^ while others did not^10,18,19^. Then, one meta-analysis published in 2017 confirmed obesity as risk for postoperative wound complication^20^. Further comparison were either limited by the lack of specific definitions of postoperative complications after reduction mammoplasty in general^13^ or a different set of predefined risk factors or complications in studies. In a meta-analysis of 10,593 patients age, smoking, BMI, radiation therapy and RTW were analyzed and a significant correlation found between smoking and postoperative wound healing disorders, as well as a high BMI and smoking increased the risk for developing postoperative complications in general^21^. This is in agreement with three other analyses, one looking at 13,503 cases from the American College of Surgeons National Surgical Quality Improvement Program^22^ and the other two in 71 and 173 cases in single center settings, respectively^19,23^. To critically evaluate data is to attribute this to the surgical technique applied: while one literature source^24^ reported no significant differences in complications comparing a vertical to an inferior pedicle technique, our data tends to come to the same conclusion, but just in comparison to a superior (cranial) pedicle technique, which is a result of either institutional or surgeon-based preference in either institutions.

Based on a complication-focused approach, the same findings apply to infection where a high BMI can be a risk factor for postoperative INF after RM^25,26^, or not on INF but on wound healing^27^. This holds true for fat tissue necrosis, where we found similar complication rates as in other recent publications of reduction mammaplasties^28,29^. Again some authors were in favor of the amount of resected tissue and age being a risk factor^30^, others against age as contributing risk factor^31^.

Our finding of allergies in the past medical history serving as significant risk factor for INF after RM, however, is quite unique in the medical literature. We can only hypothesize two possible main causes: first, patients with a history of atopy, especially atopic dermatitis, have a higher skin colonization of Staphylococcus aureus and a decreased recruitment of immune cells, which leads to a higher rate of skin infections^32–35^. And a second reason can be that a T- cell mediated, delayed type IV hypersensitivity, may be induced by antiseptics, gloves, sutures or topical skin adhesives^36–38^. Another intraoperative factor, not necessarily linked to the past medical history is surgeon experience in the case of foreign body reactions. In a retrospective review of 1000 outpatient cases in various plastic surgery operations of Gabrielli *et al*.^39^ older age was associated with comparable local tissue reactions to suture material, occurring overall in 12% of all analysed cases. Gabrielli *et al*.^39^ further gauged the experience with each surgeon in years and found significant impact on the occurrence of such foreign body reactions and wound dehiscence, concluding that foreign body reactions were preventable. Based on our data, where all operations were performed in a team of an attending plastic surgeon and a resident, we were unable to stratify data in the same way, and can neither confirm nor exclude a possible same conclusion in our data set.

Common ground in reduction mammaplasties can be found for the use of single dose antibiotics perioperatively forming part of established recommendations to reduce complications^40,41^ and supporting our finding of reducing postoperative complications, eg. in the case of wound healing problems by 57%.

Information retained by patients during informed consent for elective surgery varies and agreement on the use of information sheets is discussed without a definite conclusion based on evidence-based research^42–44^. Information supplied to a patient may be understood, but it can be easily and quickly forgotten. In an increasingly medico-legal environment, the retainment of the most common complications based on individual risk factors *and* institutional data forms a novel approach to informed consent. The aim of our provided chart is focused on the simplified explanation of the four most common complications encountered at the very institution, where the operation takes place. Whether or not this improves the retainment of information in patients is another research question. Most importantly, as long as regional/national registries fail to capture all information specific for informed consent, as well as definitions for complications fail to be analysed in a unique manner (eg. by the use of the Clavien-Dindo classification), single institutions or surgeons can either rely on varying datasets from the literature or subjective perceptions of “own” complication rates, or develop their own institutionalized and personalized tool as suggested here.

## Conclusion

Depending on the availability of data, different risk factors for different patients at different institutions apply. We present a unique patient chart to suggest a future institutionalized and personalized model-based risk profiling for optimized informed consent for elective in-patient mammaplasty. Whether this approach has any effect on patient information retainment, incidence of filed lawsuits or settlements as well as change in behaviour needs to be prospectively tested in future studies.
